# Combining multiscale niche modeling, landscape connectivity, and gap analysis to prioritize habitats for conservation of striped hyaena *(Hyaena hyaena*)

**DOI:** 10.1371/journal.pone.0260807

**Published:** 2022-02-10

**Authors:** Sahar Rezaei, Alireza Mohammadi, Shima Malakoutikhah, Rasoul Khosravi

**Affiliations:** 1 Department of Biological Sciences, Faculty of Science Engineering, University of Arkansas, Fayetteville, AR, United States of America; 2 Department of Environmental Science and Engineering, Faculty of Natural Resources, University of Jiroft, Jiroft, Iran; 3 Department of Environmental science, Faculty of Natural resources, Isfahan University of Technology, Isfahan, Iran; 4 Department of Natural Resources and Environmental Engineering, College of Agriculture, Shiraz University, Shiraz, Iran; Universidad Miguel Hernandez de Elche, SPAIN

## Abstract

Identifying spatial gaps in conservation networks requires information on species-environment relationships, and prioritization of habitats and corridors. We combined multi-extent niche modeling, landscape connectivity, and gap analysis to investigate scale-dependent environmental relationships, and identify core habitats and corridors for a little-known carnivore in Iran, the striped hyaena (*Hyaena hyaena)*. This species is threatened in Iran by road vehicle collisions and direct killing. Therefore, understanding the factors that affect its habitat suitability, spatial pattern of distribution, and connectivity among them are prerequisite steps to delineate strategies aiming at human-striped hyaena co-existence. The results showed that the highest predictive power and extent of habitats was obtained at the extent sizes of 4 and 2 km, respectively. Also, connectivity analysis revealed that the extent and number of core habitats and corridors changed with increasing dispersal distance, and approximately 21% of the landscape was found to support corridors. The results of gap analysis showed that 15–17% of the core habitats overlapped with conservation areas. Given the body size of the species, its mobility, and lack of significant habitat specialization we conclude that this species would be more strongly influenced by changes in habitat amount rather than landscape configuration. Our approach showed that the scale of variables and dispersal ability must be accounted for in conservation efforts to prioritize habitats and corridors, and designing conservation areas. Our results could facilitate the conservation of striped hyaena through the identification and prioritization of habitats, establishment of conservation areas, and mitigating conflicts in corridors.

## Introduction

Habitat loss and fragmentation can lead to patch isolation, impede individual movements, diminish suitability of the remaining habitats, and increase genetic drift and inbreeding in isolated populations [1], which can result in local extinction of species [2]. In a fragmented landscape, the long-term persistence of species is dependent on gene flow and demographic exchange between subpopulations [3, 4]. Therefore, maintaining landscape permeability has increasingly become a focus of conservation efforts, particularly for species requiring large areas and which disperse over broad extents [5]. Maintaining landscape connectivity is particularly essential for the conservation of carnivores because of their specific biological traits such as large body sizes, large home ranges, low densities, and slow population growth rates [6]. As a result, there is a need to identify spatial priorities for carnivore conservation while accounting for habitat connectivity [6–8].

Landscape connectivity modeling provides practical tools for developing conservation strategies by prioritizing habitats and corridors [4, 9]. Different methods have been proposed for connectivity modeling including resistance kernel [10], factorial least-cost path [11], Dijkstra’s shortest path [12], graph theory and connectivity metrics [13], circuit theory [14], and randomized shortest path algorithm [15]. The resistant kernel approach is one of the most widely used methods for identifying core habitats and corridors [5, 16] for amphibians [17], reptiles [1], birds [1], and mammals [18, 19]. To date, the resistant kernel has been used as a comprehensive assessment approach of connectivity for several species of carnivores [9, 19, 20]. One of the main strengths of this approach is its ability to account for the dispersal ability of focal species, the nature of dispersal function, and landscape resistance in predicting connectivity [10]. This method is a spatially synoptic approach that produces spatially explicit predictions of movement rates throughout the landscape, rather than for a few occurrence points or destination habitat patches [10]. The factorial least-cost path analysis complements the results of resistant kernel analysis by providing useful data on the most important corridors across the landscape that connect the full network of source points at a specified dispersal distance [21].

Connectivity conservation requires an understanding of the ecological variables that determine species distribution and movement patterns [5]. In this regard, ecological niche models (ENMs) have contributed to predicting the geographic ranges of many species [22]. In most published ENMs studies, spatial prediction has been produced based on environmental variables at a single spatial scale [23–26]. However, it is increasingly acknowledged that the biological, ecological, geographical, and anthropogenic processes that drive species distributions occur at multiple spatial scales [27, 28]. As a result, species responses to environmental variables, and thus their distributions, may vary across different scales [29, 30]. Therefore, predicting species distributions across multiple spatial scales is important to more accurately assess species environment relationships [8, 31, 32].

Another advantage of multiscale modeling is that by considering a range of dispersal thresholds in connectivity modeling, the analysis can assess the sensitivity of definitions of core habitats and corridors to species vagility [2, 17, 33]. Although numerous studies have used multi-scale habitat modeling and landscape connectivity separately for different species [19, 30, 32, 33], few studies have integrated the results of these analysis to predict critical habitats and corridors while accounting for the spatial scale of variables and species dispersal abilities [19, 34, 35]. Evaluating the sensitivity of results to spatial scale of analysis and dispersal ability can also provide significant insights into identifying spatial gaps in the existing network of conservation areas (CAs).

Large carnivores live at low densities and typically require large and well connected habitats [36]. Striped hyaenas (*Hyaena hyaena*) are one of the least studied large carnivores in Iran and there is limited data available on their habitat needs and spatial distribution [37]. The striped hyaena population in Iran is threatened by road vehicle collisions and direct killing (poisoning) due to perceived risk posed to pastoralists and their use in traditional medicine [37, 38]. Therefore, understanding the factors affecting their habitat suitability, spatial distribution of striped hyaenas critical habitats, and connectivity among them are prerequisite steps to delineate management strategies aiming at human-striped hyaena co-existence [39]. This species can be considered as a surrogate species and identifying core habitats and connectivity network can help in locating new reserves and protecting other co-existence species [40].

In this study, we combined the results of multi-extent ENMs and landscape connectivity models to (i) assess the influence of spatial scale of variables (defined here as extent of focal analysis) on the predicted distribution of striped hyaena, model performance, contribution of variables, and habitat composition and configuration, (ii) identify core habitats and corridors connecting habitats considering uncertainty regarding the species’ dispersal ability, (iii) rank core habitats based on their characteristics and role in facilitating species movements, and (iv) evaluate the coverage of existing CAs in protecting core habitats and corridors using gap analysis.

## Methods

### Study area

**Fig 1:**
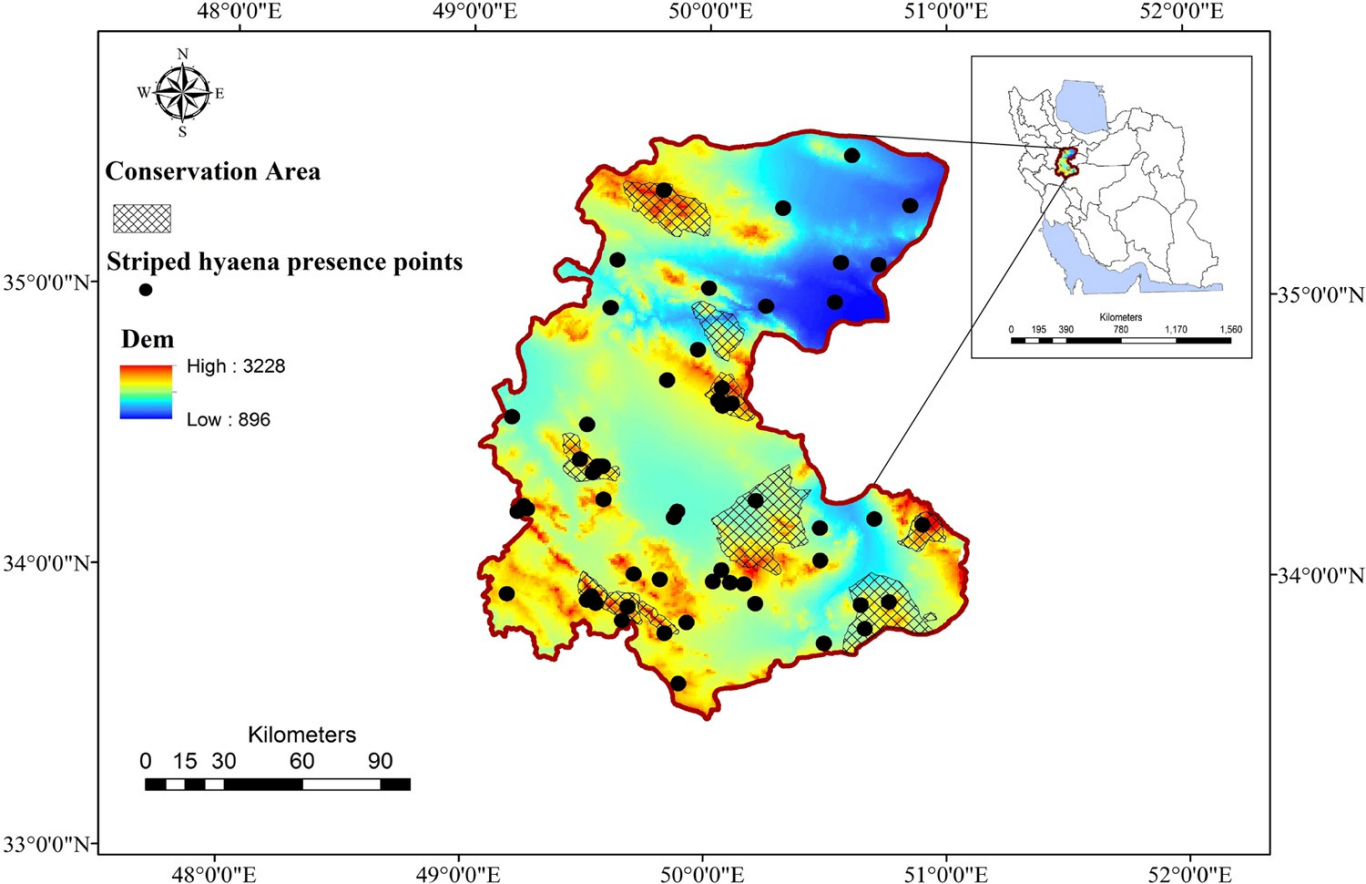
Location of the study area used to perform multi-extent distribution modeling and landscape connectivity for striped hyaena in central Iran. Republished from data provided by Markazi Provincial Office of Department of Environment (MDoE) under a CC BY license, with permission from MDoE, original copyright, 2016.

The study was conducted across a part of the striped hyaenas range in the Markazi province located in the central Iran (33°30 to 30°535 N; 48°57’ to 57°51’ E; Fig 1). This area is 29,127 km^2^ in extent and is bounded between the central desert and the collision point of Alborz and Zagros faults. Despite the arid and semi-arid environmental conditions, this part of Iran supports a high diversity of large and medium-sized carnivores, including grey wolf (*Canis lupus)*, golden jackal (*Canis aureus*), red fox (*Vulpes vulpes*), African wildcat (*Felis lybica*), Persian leopard (*Panthera pardus saxicolor*), and caracal (*Caracal caracal*; [41]). The region also supports three ungulate species, including mouflon (*Ovis gmelini*), goitered gazelle (*Gazella subguturosa*), and wild goat (*Capra aegagrus*). The dominant vegetation types include the *Artemisia spp*, *Scariola orientalis*, *Astragalus spp*, and *Euphorbia sp* [42]. There are two wildlife refuges (WRs; IUCN category IV; 2322.01 km^2^), two protected areas (PAs; IUCN category V; 1366.47 km^2^), and five non-hunting areas (NHAs; no IUCN category; 1315.01 km^2^) for protecting biodiversity [42].

### Striped hyaena presence data and environmental variables

The occurrence localities for striped hyaena were obtained from a variety of sources including observations of denning sites (n = 55), scat identification (n = 88), and opportunistic sightings (n = 45) from 2017 to 2019. Hyaena scats were identified by shape, size, and colour [43, 44]. To evaluate the spatial autocorrelation of the occurrence data, we calculated global Moran’s *I* using ArcGIS v10.2 [45], which showed that the occurrence points were spatially uncorrelated (S1 Fig).

We selected ten environmental and anthropogenic variables likely to affect species’ distribution based on their relevance to the species’ ecology [37, 39, 46]. A digital elevation model (DEM) from the 90 m Shuttle Radar Topography Mission (http://earthexplorer.usgs.gov) [47] was used to calculate slope and topographic roughness using the Spatial Analyst Toolbox, and Geomorphometry and Gradient Metrics extensions, respectively [48] in ArcGIS. To calculate the normalized difference vegetation index (NDVI), we extracted red and near infrared bands (http://earthexplorer.usgs.gov/) for the year 2016 at 30 m resolution and calculated the index using the Image Analysis tool in ArcGIS. We extracted vegetation types with coverage exceeding 25% from the landcover map of the study area (personal communication with the Markazi Department of Environment, 2016) and calculated the density of the extracted vegetation types within a window of 1 × 1 km using the Neighborhood statistic tool. Given our expectation that distance to human disturbances and agriculture would be meaningful predictors of the species occurrence, we calculated the Euclidean distance to croplands, roads, human settlements, and dumpsites using the Spatial Analyst Toolbox in ArcGIS (S1 Table).

Prey availability was obtained by combining habitat suitability and relative abundance maps of three wild ungulate species including wild goat, mouflon, and goitered gazelle [29]. We obtained data on the occurrence localities of three main prey species from Markazi Department of Environment between 2015 and 2018 and also from presence data used in the study of Karami et al. 2020 [49]. We modelled the distribution of each prey species using the maximum entropy algorithm, MaxEnt, [50] (see S1 Text for details on the process of prey distribution modeling). To estimate prey density, we used data on prey species abundances obtained within conservation areas and unprotected areas across the region. For each prey species, we divided the abundances in each conservation area/ unprotected landscape by their areas and assigned the estimated density values to all the associated pixels. In the final step, the habitat suitability and density maps for each species were multiplied and summed to estimate prey availability. We prepared all the layers at a spatial resolution of 100 m. To avoid multicollinearity among variables, we calculated Pearson’s correlation coefficient and removed variables with a Pearson’s correlation greater than 0.8 [18, 51].

### Spatial niche modeling

Because different predictors may be related to habitat selection of species at different scales [52], the spatial distribution of striped hyaena was modelled at five different extent sizes of variables (0.1, 0.5, 1, 2, and 4 km) to span from local foraging (400 m/hr) to the size of the species’ home range (urban regions (~6 km^2^) and rural (56 km^2^) [46, 53, 54]. To implement our modeling approach, we calculated the mean values of each variable using a moving window with a circular neighborhood corresponding to each extent size described above in ArcGIS. All MaxEnt models were run with ten replicates, 10000 background points, 500 iterations, and bootstrap replicated run type. We used 75% of occurrence points to calibrate the models and the remaining 25% to evaluate model predictions [31, 32]. To evaluate the performance of models, we calculated the area under the receiving operating characteristic curve (AUC). To further compare models across the five extent sizes, specifically in terms of area of suitable habitats, we converted the continuous suitability maps to suitable/unsuitable habitats using the 10^th^ percentile training presence [55], and the mean suitability values at the occurrence points [18] as thresholds.

### Effects of extent sizes of variables on the area and connectivity of suitable habitats

We assessed the effect of extent sizes of variables on the area and degree of connectivity of predicted suitable habitats by calculating four landscape metrics including the percentage of landscape covered by suitable habitats (PLAND), largest suitable patch index (LPI), correlation length of suitable habitat patches (CL), and number habitat patches (NP) for each binary map [56–58] using FRAGSTATS [59]. Correlation length is the expected distance an individual can move in a random direction from a random starting locality within a habitat patch before encountering the patch boundary. Largest patch index is the size of the largest connected habitat patch as a proportion of the extent of the study landscape.

### Connectivity corridor network simulation

To estimate landscape movement resistance, we converted the habitat suitability map at the best extent size of variables (based on AUC) to a resistance surface using an exponential decay function (R = 1000^(-1×HS)^), where R represents the cost resistance values assigned to each pixel and HS represents the predicted suitability [60, 61]. We rescaled the resistance values to a range between 1 and 10 by linear interpolation, such that minimum resistance was 1 when HS was 1 and maximum resistance was 10 when HS was 0 [62].

We employed the universal corridor network simulator, UNICOR [12], to predict core habitats and corridors. UNICOR’s key features include a driver-module framework, connectivity mapping with thresholding and buffering, and graph theory metrics. The factorial least-cost path analysis [11] implemented in UNICOR relies on Dijkstra’s algorithm [12]. The analysis produces predicted least-cost path routes between each pair of occurrence locations. The resistant kernel algorithm [10] calculates the resistance cost weighted dispersal kernel around each source point up to a user-defined dispersal threshold and sums these to produce an incidence function of the rate of species movement through every pixel in the landscape as a function of the number and density of source points, the species’ dispersal ability, and the resistance of the landscape [63].

The maximum dispersal rate of species is dependent on different factors such as resource availability, seasonal patterns, habitat suitability, landscape composition and configuration, species’ behavior, and demographic variables [19]. According experts consulted in this study, the mean distance walked of the species is around 400 m/hr in urban areas with the longest distance recorded 1 km/hr and 800 m/hr in rural areas with the longest distance of 2 km/hr (Mounir R. Abi-Said, pers. communication). To account for uncertainties regarding movement behavior and reliable dispersal data for striped hyaena in Iran and evaluate how robust our predicted core habitats are to this uncertainty, we ran a sensitivity analysis. Hence, we analyzed five dispersal thresholds in the resistant kernel, including 50000, 100000, 150000, 200000, and 250000 cost units, which represent maximum movement abilities through optimal habitat (resistance value of 1) of 50, 100, 150, 200 and 250 km, respectively [2]. Using these ranges of dispersal rates allows us to cope with such lack of data about dispersal movement of the species while producing conservative estimates. We calculated the factorial least-cost path network without a dispersal threshold to provide a broad-scale assessment of the regional pattern of potential linkage and to map corridors [1]. The buffered least-cost paths were then combined through summation [11] to produce maps of connectivity among all pairs of presence points.

### Conservation prioritization and gap analysis of core habitats and conservation areas

We defined core habitats as contiguous units with resistant kernel values >70^th^ percentile of the predicted resistance kernel for the species [7]. Then we applied a gap analysis [64] and a graph network algorithm [65] to assess the relative importance of the predicted core habitats to landscape connectivity and prioritized them. Specifically, we calculated delta probability of connectivity (dPC; [66]) as recommended by Saura and Torne (2009) [67] for prioritizing predicted core patches at the six movement ability scenarios in Conefor 2.2 [67]. The dPC depends on the inter-patch dispersal distance and the predicted kernel values; it uses graph theory to evaluate the importance of each patch based on its quality and connectivity in the full network of other core patches. We calculated the dPC based on two characteristics of core patches including patch’ extent [8, 16, 68] and patch’ strength (sum of kernel values in the patch). To measure the patch’s strength [7], we overlaid the shapefiles of the predicted core patches at each dispersal scenario with the predicted kernel surfaces and calculated the sum of kernel values within the patch boundaries. Also, we used Euclidean distance between each pair of core habitats as a connection file. We also used gap analysis to evaluate the sufficiency of the existing conservation network. We assessed species representation for three categories of conservation areas (wildlife refuge, protected area, and non-hunting area) by calculating dPC [69]. We examined three fractions of the dPC (intra, flux, and connector) to gain further insight into the relevance of each conservation areas for connectivity. The dPC-intra only considers intrapatch connectivity, whereas dPC-flux considers both patch features (e.g., the extent of suitable habitats) and its location, and dPC-connector only considers patch’s topological position in the landscape [35, 70]. We assumed that each conservation area functioned as a habitat patch and used the extent of suitable habitats within them as patch attributes for calculating PC values [67].

## Results

### Spatial distribution of striped hyaena

**Fig 2:**
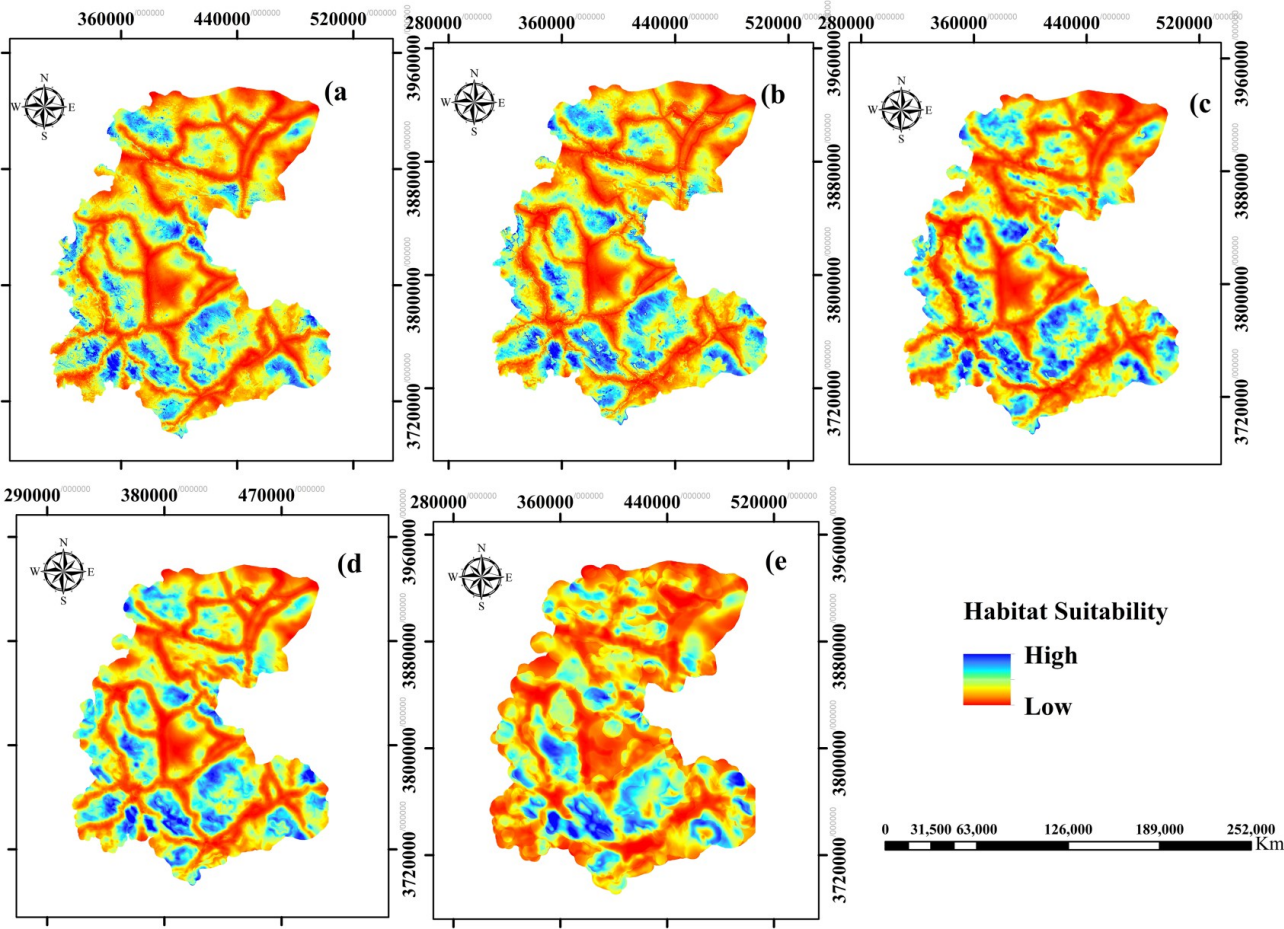
Habitat suitability of striped hyaena in central Iran at five extent sizes of variables including 0.1 (a), 0.5 (b), 1 (c), 2 (d), and 4 km (e). Republished from data provided by Markazi Provincial Office of Department of Environment (MDoE) under a CC BY license, with permission from MDoE, original copyright, 2016.

**Fig 3:**
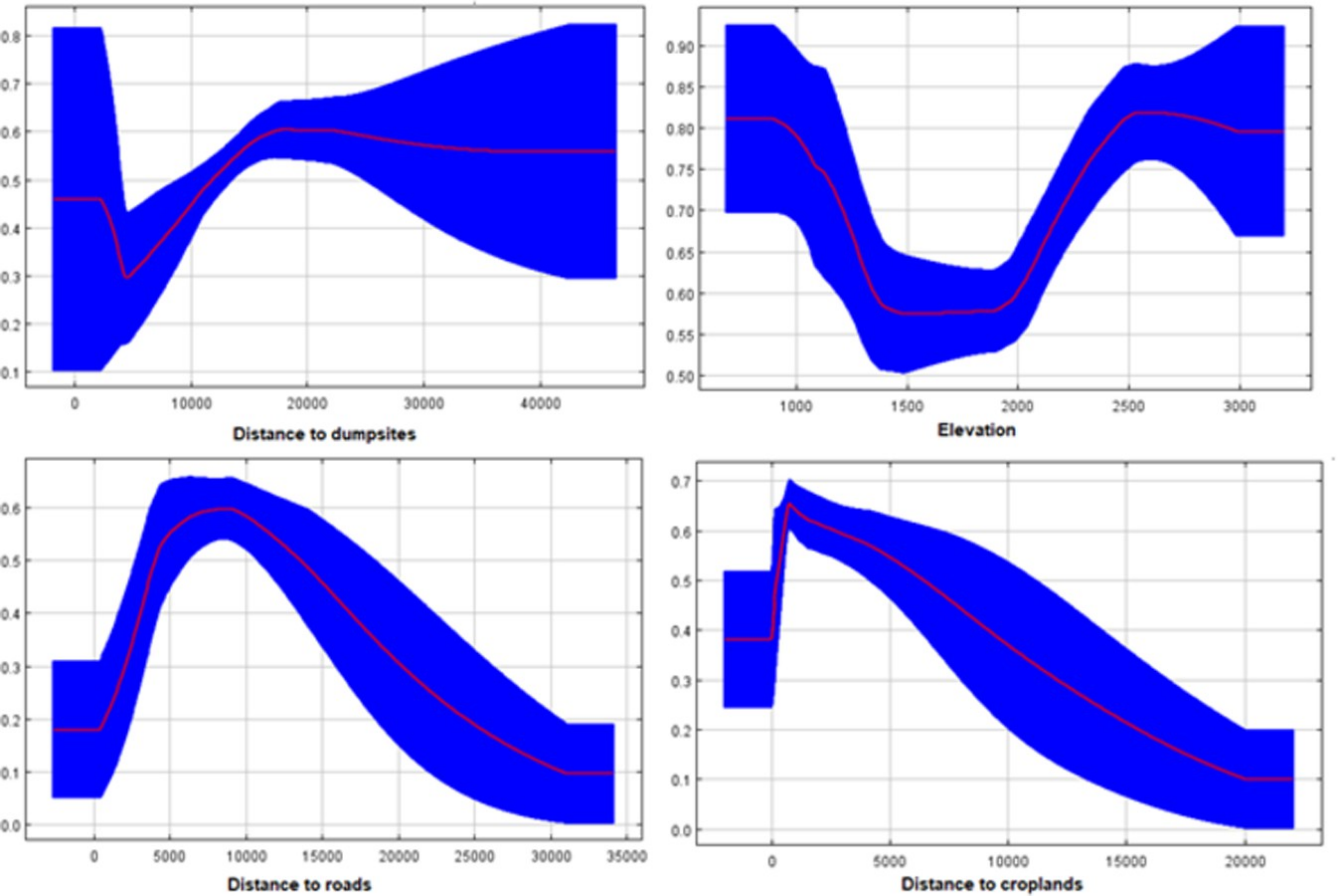
Response curves of the most influential predictors for distribution of striped hyaena at the extent size of 4 km in central Iran.

While all models showed high predictive performance (AUC > 0.8), the predicted spatial distributions at the five extent sizes of variables showed spatially different patterns (Fig 2). The extent sizes of 4 and 0.1 km had the highest and lowest performance in predicting suitable habitats of striped hyaena, respectively; however, the differences were slight (Table 1). Distance to roads, elevation, distance to croplands, and distance to dumpsites had the highest contributions in determining striped hyaena habitat suitability. However, the three latter variables represented different rankings across the different extent sizes (S2 Text and S2 Fig). Moreover, the response curves of the best models (4 km) showed that distance to croplands, roads, dumpsites, and elevation play crucial roles in the species’ distribution (Fig 3).

**Table 1:** Model performance, importance of variables, area of suitable habitats, and coverage of conservation areas obtained for the stripped hyaena in central Iran according to spatial niche predictions at five variable extent sizes.

Extent size (km)	Top most important variables	AUC	Suitable habitats (km^2^)		Coverage of conservation areas (%)	
			Threshold for suitable habitats			
			Mean	10^th^ percentile	Mean	10^th^ percentile
0.1	distance to roads, distance to croplands, distance to dumpsites, topographic roughness	0.84	3488.49	14664.54	37	16
0.5	distance to roads, elevation, distance to croplands, prey availability	0.86	3796.29	14784.26	34	19
1	distance to roads, elevation, distance to croplands, distance to dumpsites	0.86	4107.05	14274.51	29	19
2	distance to roads, elevation, distance to dumpsites, topographic roughness	0.85	3920.46	1554.150	32	17
4	distance to roads, elevation, prey availability, distance to dumpsites	0.88	3776.35	15091.96	30	18

The area of suitable habitats varied across the five extent sizes of variables and suitability thresholds (S3 and S4 Figs). The minimum and maximum area, considering the mean suitability value at the occurrence points as threshold, was obtained at the extent sizes of 0.1 and 1 km, respectively. However, for the threshold of 10^th^ percentile training presence, the minimum and maximum area were predicted at the extent sizes of 1 and 2 km, respectively (Table 1).

Similar scale-dependent patterns were observed for the coverage of the conservation areas in providing suitable habitats (Table 1). On average, the conservation areas overlapped with 32.4% and 17.8% of the predicted suitable habitats for the mean and 10^th^ percentile training presence thresholds, respectively. At the 10^th^ percentile training presence, non-hunting areas provided the most coverage of predicted habitats at all extent sizes of variables. However, for the mean threshold value, non-hunting areas had the highest coverage at extent sizes of 0.1, 0.5, and 4 km, and protected areas at 1 and 2 km (S2 Table in Resource Online 1).

### Composition and configuration of suitable habitat patches

**Fig 4:**
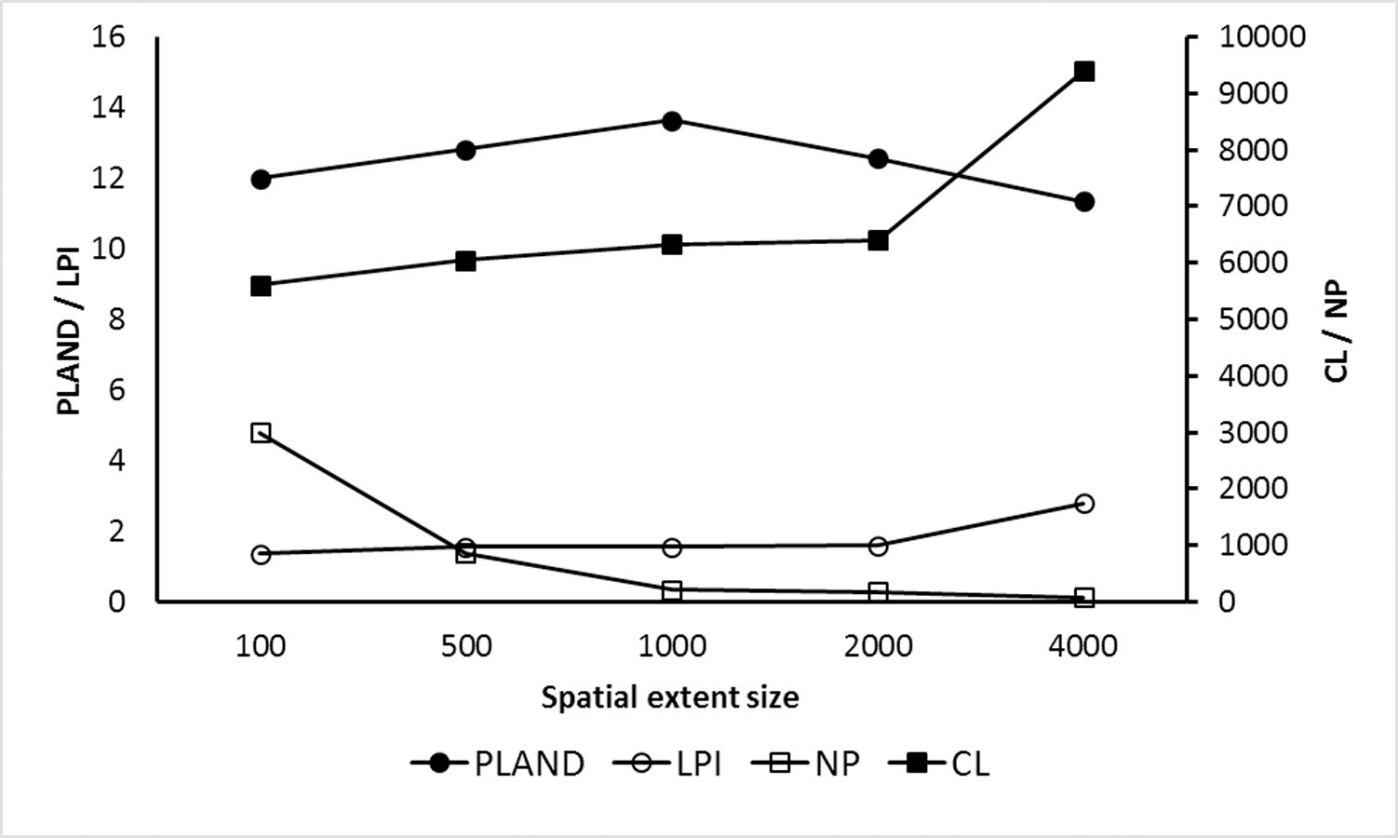
Graph of four landscape composition and configuration metrics to evaluate connectivity between suitable habitats of striped hyaena across the five extent sizes in central Iran.

The extent size of predictors had substantial effects on the composition and configuration of the predicted suitable habitats (Fig 4). The result shows that the importance of the configuration metrics is higher than composition in suitable habitats of striped hyaena across the five extent sizes. The percentage of the landscape covered by highly suitable habitats (PLAND) was predicted to substantially increase between the extents of 0.1 and 1 km and then decreased at the broadest extent size. The correlation length (CL) and largest patch index (LPI) of suitable habitats were predicted to increase with increasing scale of analysis. Both metrics slightly increased across the extent size of 0.1 to 0.2 km. However, these measures increased rapidly between 2 and 4 km. In contrast to increase in the LPI and CL, the number of patches of suitable habitats substantially decreased across the five extent sizes and this decline had a strong nonlinear threshold between the extents of 0.1 and 0.5 km.

### Core habitats and corridors

**Fig 5:**
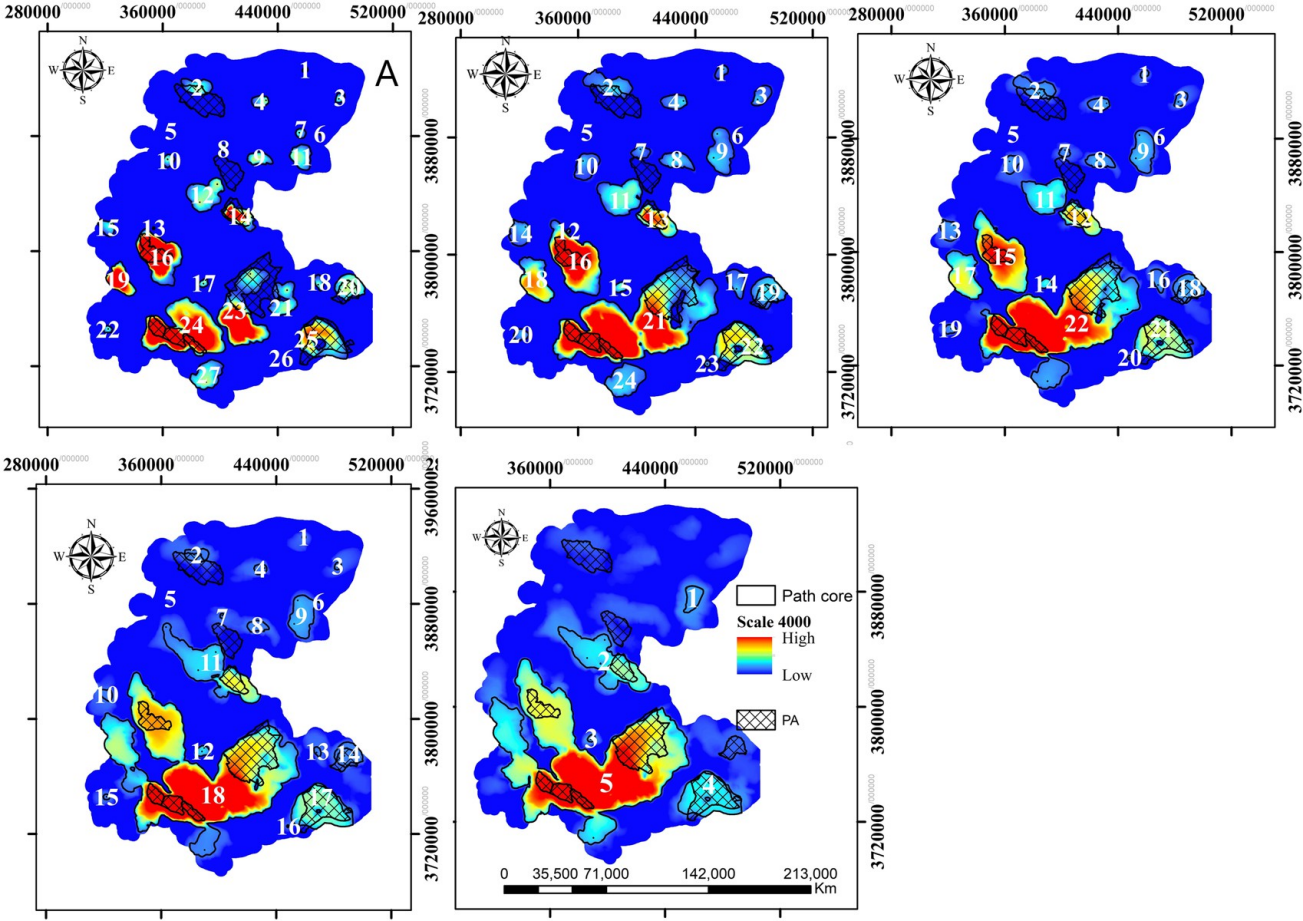
Predicted core habitats at variable extent size of 4 km and five dispersal scenarios including 50 (a), 100 (b), 150 (c), 200 (d) and 250 (e) km. Republished from data provided by Markazi Provincial Office of Department of Environment (MDoE) under a CC BY license, with permission from MDoE, original copyright, 2016.

Our connectivity simulation at the extent size of 4 km (the best performing spatial distribution model) revealed that the area and number of predicted core habitats changed rapidly with increasing dispersal distance (Fig 5; S3 Text). Across dispersal distances, most of the large core patches were in the southern part of the landscape. Furthermore, we predicted many core habitats outside the existing CAs (Fig 5).

We delineated six corridor networks linking core habitats, all of which were larger than 50163.77 km^2^ (S5 Fig). Approximately 21% of the landscape was predicted to be suitable for movement of striped hyaena, with varying degrees of connectivity strength. At all dispersal distances, the most important predicted corridor network, with regards to the strength of the corridor and the connected core areas, were concentrated in soutern and central parts of the lanscape, linking the three most important core areas.

The contribution of the predicted core habitats to landscape connectivity revealed a different pattern of ranking depending on patch characteristics (patch size and patch strength) and dispersal distance (Table 2). Also, we found a high variability in dPC of the predicted patches at each dispersal distance. Furthermore, increasing patch area and patch strength led to increasing values of the dPC. Also, for both patch characteristics, dPC values tended to increase as dispersal distance increased (S6 Fig).

**Table 2:** Mean values of delta probability of connectivity (dPC) calculated for the predicted core habitats at five dispersal scenarios in central Iran. The numbers in parenthesis show the core habitat numbers.

Rank	Patch area						Patch strength			
	Dispersal distance (km)						Dispersal distance (km)			
	50	100	150	200	250	50	100	150	200	250
1	45.61 (24)	76.56 (21)	78.52 (22)	93.29 (18)	94.94 (5)	58.09 (24)	87.04 (21)	89.23 (22)	98.54 (18)	98.92 (5)
2	26.85 (23)	14.31 (16)	18.49 (15)	13.07 (11)	14.48 (2)	23.41 (16)	16.74 (16)	17.99 (15)	5.73 (11)	6.29 (2)
3	13.37 (16)	6.43 (22)	8.37 (21)	7.41 (17)	8.52 (4)	19.34 (23)	3.54 (22)	4.84 (21)	3.79 (17)	4.01 (4)
4	7.77 (25)	5.52 (11)	6.83 (17)	2.33 (9)	2.30 (1)	3.87 (19)	3.29 (18)	4.10 (17)	0.57 (9)	0.62 (1)
5	4.72 (21)	5.03 (24)	6.79 (11)	1.75 (14)	0.93 (3)	2.64 (25)	2.53 (13)	3.40 (12)	0.41 (12)	0.32 (3)

The results of gap analysis showed relatively low overlap of the predicted core habitats at each dispersal distance with conservation areas. The existing conservation areas provided coverage for 15% to 17% of the predicted core habitats at different dispersal distances. Among these conservation areas, protected areas with a total extent of 106,042 km^2^ showed the highest (8.18%) coverage, followed by non-hunting areas (6%), and wildlife refuges (1.83%; Table 3).

**Table 3:** Percentage of protected core habitats covered by conservation areas at five dispersal scenarios.

Conservation area	IUCN Category	No.	Area (km2)	Conservation coverage (%)				
				Dispersal distance (km)				
				50	100	150	200	250
Wildlife refuge	Category IV	2	27856	3	2	2	2	1
Protected area	Category IV-VI	2	106042	6	8	8	9	9
Non-hunting area	Category V	5	131487	8	6	6	6	5
Total		9	265385	17	16	16	17	15

Among the conservation areas, Chal-khatoon non-hunting area showed the highest value of dPC, followed by Rasvand wildlife refuges, and Alvand protected area. Considering the three fractions of the dPC index together confirmed that Rasvand had the highest score for dPC-flux, while Chal-khatoon had the highest values for dPC-connector (S3 Table).

## Discussion

### The effect of environmental variables on the striped hyaena’s occurrence

All models developed at different extent sizes of variables predicted that habitats with the highest suitability were located relatively near roads. One possible explanation for the significant impact of roads is that roads provide supplementary and complementary food resources such as road-killed animals for the striped hyaena [71–74]. Although occurrence points were not spatially correlated, this relationship should be interpreted with caution due to the possibility of sampling bias by collecting more presence points near the roads. Croplands are an important food source for striped hyaena because these areas provide scavenging opportunities for the species on carcasses of livestock [29]. Our results indicated that striped hyaenas tend to avoid areas with the highest human activities such as dumpsites and human settlements, which suggests a threshold relationship with the intensity of anthropogenic factors where hyaenas avoid the areas with highest human activities [75–78]. In addition to human food resources, we also found that natural food resources (prey availability) play a vital role in hyaenas’ presence. A strong positive association was found between the concentration of prey species within the conservation areas and predicted suitability of these areas for striped hyaenas. This species selected high elevation areas, which is a reflection of general avoidance of human activities (other than agriculture and roads) and providing favorable conditions for den selection behaviour [37, 79].

### Effects of extent size of variables on model performance and area of suitable habitats

ENMs’ improvement through the multi-scale approach gave us a more comprehensive picture of the species’ environmental requirements in Iran, and enabled us to clarify predictions of highly suitable areas and improved descriptions of the species’ realized ecological niche [80, 81]. In addition, this method enabled us to filter the predictions according to the availability of habitats, which exist locally and also to identify areas where special attention is needed [82]. Moreover, multiscale modeling significantly boosts the accuracy of the prediction, which is highly valuable for carnivore species conservation strategies, in order to efficiently measure local vulnerabilities, prioritize areas for early detection and control, and limit their impact [80].

Striped hyaena in Iran responds to the most landscape features at relatively broad scales. Such strong correlation has also been reported by other studies on carnivores [19, 81, 83]. This relationship may be due to striped hyaenas broad avoidance of human activities in remnant habitats within human-dominated landscapes. Previous multi-scale habitat suitability studies on carnivores have suggested that large carnivores often correlate most strongly with human activities at relatively broad scales, whereas factors that affect resting and foraging typically are most important at finer scales [9, 27]. We found fine-scale sensitivity of the striped hyaena to roads, with the strongest contribution of roads observed at the 0.1 km extent size, which was in contrast to that of human settlements and dumpsites. This relationship probably reflects a tendency of the species to associate with roads for supplementary resources from scavenging roadkill.

The majority of previous studies have confirmed that landscape configuration is relatively less important than composition for large carnivores, such as Persian leopard and Asiatic cheetah [27, 68]. However, other studies have shown that in landscapes with small amounts of habitat area, landscape configuration may have large impacts on species persistence [71, 72]. Given the body size of striped hyaena, its mobility, and lack of significant habitat specialization, as well as relatively large extent of habitats across the study landscape, we conclude that this species would be more strongly influenced by changes in habitat amount (landscape composition) rather than configuration.

In addition, fragmentation of striped hyaena’s habitat (as measured by the number of isolated patches) decreases nonlinearly with increasing extent size of variables. Also, the landscape composition metric (i.e., PLAND) increased linearly between 0.1 to 1 km scales of analysis. This suggests that inferences about the area of suitable habitats and the effects of habitat fragmentation on species are highly sensitive to extent size of variables. Therefore, considering the high mobility of the species, which allows them to integrate across landscapes of differing configurations, assessing the effects of habitat fragmentation at fine extent size of variables, may overestimate the impacts habitat fragmentation has on the survival of the species.

### Connectivity of core habitats

Considering a dispersal distance of 100 or 150 km, as more reliable dispersal scenarios for the striped hyaena, our predicted connectivity suggest that although the southern and western parts of the landscape have an extensive area of connectivity, the populations of the species are divided into a number of patches that are fragmented. Strong scale dependency in connectivity predictions to dispersal ability have been widely found in past studies [2, 84]. Consistent with our findings, Ash et al. (2020) [85] suggested that parameters associated with dispersal ability and mortality risk have greater effects on the predicted connectivity, population size, and distribution of Indochinese tigers (*Panthera tigris corbetti*) in Southeast Asia than do differences in landscape resistance models [85].

In addition to dispersal ability, the functionality of the predicted corridors, especially in the southern part, may be decreased if the effects of roads and possible human-hyaena conflict within corridors is considered. Moqanaki and Cushman (2017) [86] and Khosravi et al. (2018) [18] also confirmed the role of roads as impactful fragmenting features and as a cause of vehicle-induced wildlife mortalities. Most of the conservation areas in Iran are surrounded by roads, so road mortalities are considered to be a serious threat for carnivores especially in corridors [18, 74, 86, 87]. For instance, Parchizadeh and Adibi (2019) [88] showed that road killings are among the most important human‐caused mortality factors for Persian leopard in Iran. Therefore, it is important to conserve both core areas and corridors [73, 74, 86, 89] to sustain species core populations and enable dispersal among them in the face of various threats [2, 19, 90, 91].

### Contribution and prioritization of core habitats and conservation areas to landscape connectivity

Patch importance positively correlated with the extent of core habitats (patch quantity) and patch strength (patch quality) regardless of the dispersal ability. These results are consistent with Ahmadi et al. (2017) [92] and Khosravi et al. (2017) [18] who worked on Asiatic cheetah, Persian leopard, and sand cat (*Felis margarita*). Patch quality, more than patch extent, had larger impacts on landscape connectivity in these studies. This suggests that patch extent alone may not be effective in prioritizing core habitats and, additionally, the quality of patches and species dispersal ability should be considered in management efforts to prioritize habitats.

The coverage core habitat patches within the conservation areas showed that, across extent sizes of variables and dispersal ability, less than 20% of predicted core habitats are currently protected. Therefore, for effective conservation of striped hyaena, the proportion of conservation areas should be increased and allocated optimally and strategically to maximize the protection of habitat extent and, secondarily, to maintain connectivity network. In addition, since the highest-ranked core habitats are located in the southern parts, establishing new conservation areas in this part of the landscape is of the utmost importance to the protection of striped hyaena populations.

Establishing more strictly conservation areas is politically challenging. Therefore, we strongly recommend establishing new less strictly conservation areas, such as non-hunting areas or indigenous and community conservation areas (ICCAs), in the predicted high-ranked patches. Another alternative may be to develop networks of interconnected highly ranked core habitats. In some cases, managing and sustaining an interconnected network of core habitats may be more viable than establishing new conservation areas [93].

While designating new conservation areas is often politically difficult [94], an alternative may be to develop networks of interconnected highly ranked core habitats. In some cases, managing and sustaining an interconnected network of core habitats may be more viable than establishing new protected areas [93]. These networks of connected core patches could aid gene flow and prevent isolation of small populations [85]. Establishing new conservation areas is recognized as a critical component of conservation strategies [2, 77]. Nevertheless, for conservation areas to be effective in promoting conservation objectives they need to be carefully selected to provide sufficient conditions, extent, and connectivity to sustain the biodiversity that depends on them [18]. A study by Cushman et al. (2016) [2] showed that strictly conservation areas are essential as the foundation of conservation strategies for large carnivores, but are insufficient in terms of their current network, requiring both expansion of existing conservation areas and strategic protection of critical linkage corridors among them. Therefore, considering both representativeness and spatial configuration of the predicted core habitats in the process of selection of new less strictly conservation areas, would decrease the main biases in existing networks.

Connectivity and gap analysis varies depending on the attributes of the target species (i.e. dispersal distance) and scale of the research [69, 95]. Based on dPC-connector, non-hunting areas provide a greater contribution to network connectivity than other conservation units. Consistent with our results, Khosravi et al. 2018 [18] also emphasized the importance of this category of conservation areas, as stepping stones, for large carnivores habitat connectivity. Moreover, Ahmadi et al. (2020) [69] showed that non-hunting areas had the highest priorities for habitat connectivity and population integrity of Asiatic cheetah and Persian leopard in Iran.

### Model limitations and future directions

It should also be noted that findings of connectivity studies based on presence points and habitat models are limited by uncertainties [60, 96]. Specifically, our analysis used a relatively modest sample of presence-only data, which could limit the power of our predictions. However, the high performance of our models using bootstrapped and cross-validated model assessment suggests that our habitat predictions are robust. The studied landscape was only part of the current range of striped hyaena in Iran and we did not include the whole distribution range of the species in our analysis. It would be better to fit connectivity models with movement [97] or gene flow [93] data instead of habitat models, as dispersal is often related to different factors at different scales than home range. Therefore, satellite tracking [97] and landscape genetic studies are also necessary to make more reliable predictions and validate the findings. To conclude, habitat suitability and connectivity models should be considered as the first step towards building a nation-wide strategy for corridor improvement [98].

### Implications for conservation

Habitat requirements and high movement ability of the striped hyaena emphasizes that long-term conservation planning for this species should cover its conservation requirements at broad spatial scales through protection of a network of core habitats connected by corridors [99]. The obtained results could be useful and informative in two ways. First, spatial predictions of core habitats and corridors identify areas of high conservation value as priority areas for allocation of the limited resources for conservation of the striped hyaena. Second, these results could be integrated into land use plans designed to prevent /minimize negative impacts of anthropogenic activities on the core habitats and corridors that would remain unprotected.

Mitigation of human-hyaena conflicts in corridors to increase the functionality of the corridors is one of the most important conservation implications of the study. Apart from positive function of corridors in enhancing connectivity, they can also potentially increase potential human-carnivore conflicts by promoting movement into areas with high risk of human encounter. Therefore, if we do not pay attention to human-carnivore conflicts when identifying corridors, it may lead to limiting the dispersal of individuals into ecological traps and overvaluing the effectiveness of corridors for carnivore movement [100]. In addition, large carnivores in human-dominated landscapes often cannot persist inside conservation areas alone and critically depend on habitat outside protection areas. Ghoddousi et al. 2020 [100] showed that many conservation areas are not large enough to provide suitable habitat for carnivores. The predicted unprotected core habitats can be considered as potential areas to establish new less strictly conservation.

## Supporting information

S1 Fig The results of global Moran’s I to evaluate the spatial autocorrelation in the occurrence localities of striped hyaena in central Iran. (DOCX)

S2 Fig Response curves of the most influential predictors for distribution of striped hyaena in central Iran at different extent sizes of variables (0.1, 0.5, 1, 2, and 4 km). (DOCX)

S3 Fig Binary habitat suitability maps for striped hyaena in central Iran at extent size of 0.1 (a), 0.5 (b), 1 (c), 2 (d) and 4 km (e) using the mean suitability score at occurrence points as threshold.
(DOCX)

S4 Fig Binary habitat suitability maps for striped hyaena in central Iran at extent size of 0.1 (a), 0.5 (b), 1 (c), 2 (d) and 4 km (e) using the 10-percentile training presence threshold.
(DOCX)

S5 Fig Predicted corridors’ network linking striped hyaena core habitats and conservation areas at variable extent size of 4 km in central Iran. The colour gradient represents predicted connectivity from weak (blue) to strong (red).
(DOCX)

S6 Fig Mean values of delta probability of connectivity (dPC) index calculated for the predicted core habitats of striped hyaena in central Iran based on their extent and strength at five dispersal scenarios. (DOCX)

S1 Table List of the environmental variables used to predict distribution of *H*. *hyaena* in central Iran. (DOCX)

S2 Table The area of suitable habitats (km^2^) for striped hyaena covered by the existing conservation areas at five extent sizes of variables in central Iran. (DOCX)

S3 Table The most important conservation areas ranked according to the delta probability of connectivity (PC) index and its three fractions (intra, flux, and connector) at the variable extent sizes of 4 km. (DOCX)

S4 Table The list of occurrence localities of striped hyaena *(Hyaena hyaena*) used for habitat suitability modeling. (DOCX)

S1 Text Predicting the distribution of the prey species. (DOCX)

S2 Text The impact of variables on the predicted habitat suitability of striped hyaena. (DOCX)

S3 Text The number of recognized habitat patches for different dispersal ability scenarios. (DOCX)
