# Supplementary material for: Combining multiscale niche modeling, landscape connectivity, and gap analysis to prioritize habitats for conservation of striped hyaena (Hyaena hyaena)
Source: PLoS One. 2022 Feb 10;17(2):e0260807. doi: 10.1371/journal.pone.0260807 (PMC8830629; doi:10.1371/journal.pone.0260807)
Supplement: S1 Fig — (DOCX) [file pone.0260807.s001.docx]

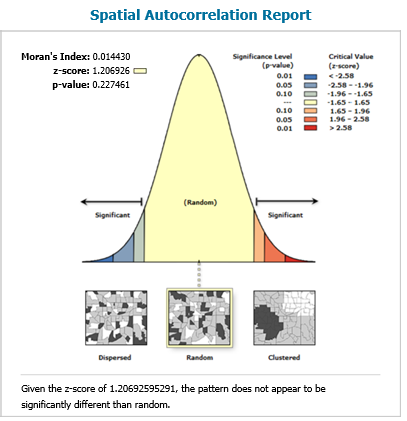

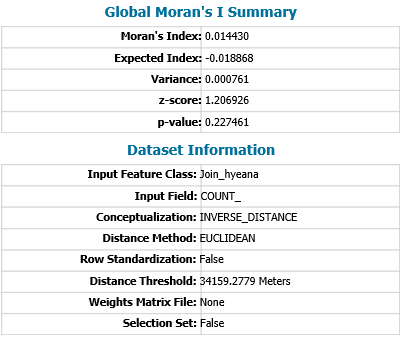


**Fig. S1**. The results of global Moran’s I to evaluate the spatial autocorrelation in the occurrence localities of striped hyaena in central Iran.
