# Supplementary material for: Combining multiscale niche modeling, landscape connectivity, and gap analysis to prioritize habitats for conservation of striped hyaena (Hyaena hyaena)
Source: PLoS One. 2022 Feb 10;17(2):e0260807. doi: 10.1371/journal.pone.0260807 (PMC8830629; doi:10.1371/journal.pone.0260807)
Supplement: S2 Fig — (DOCX) [file pone.0260807.s002.docx]

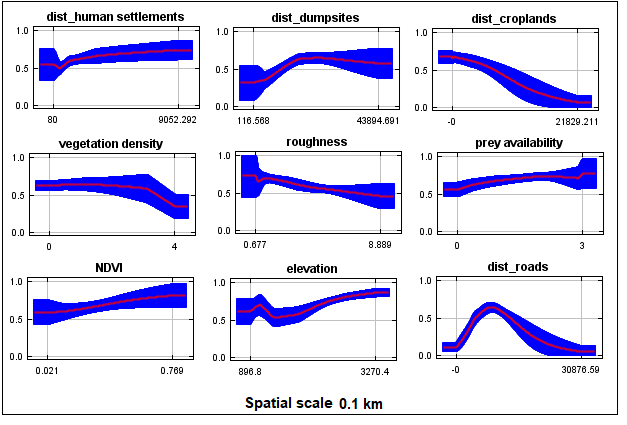


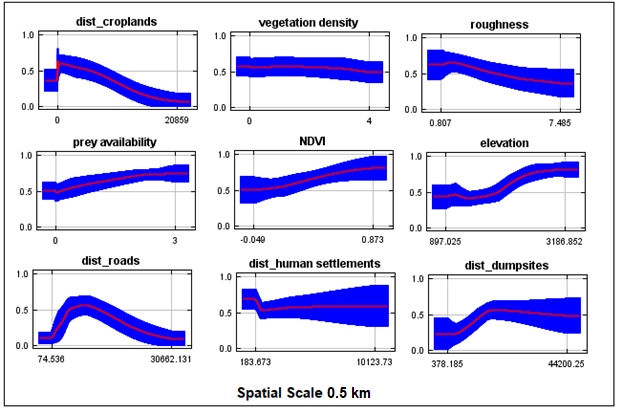


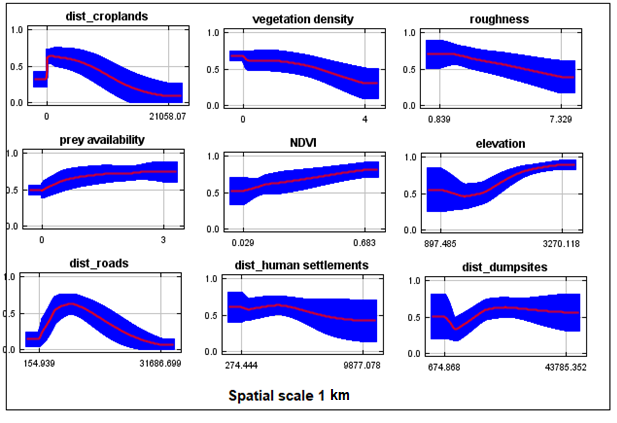


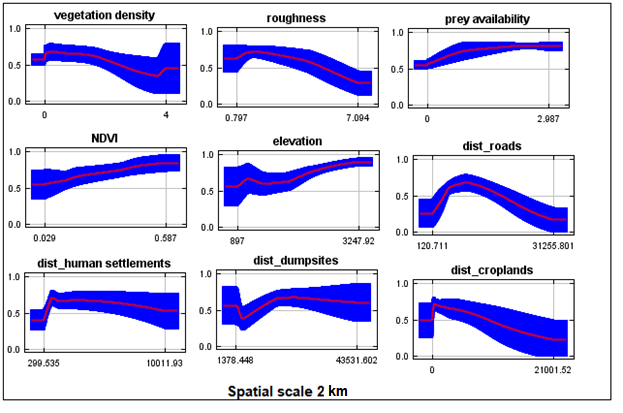


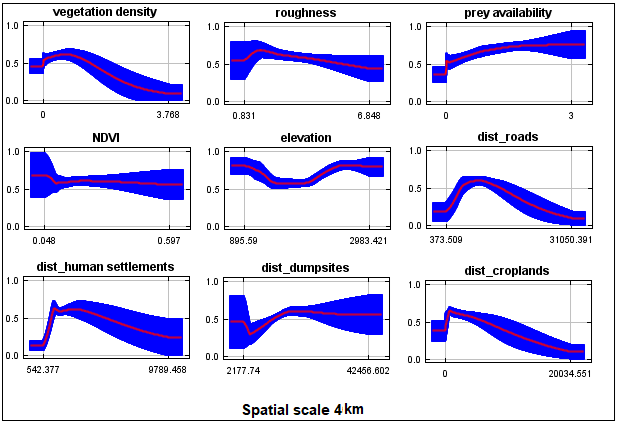


**Fig. S2**. Response curves of the most influential predictors for distribution of striped hyaena in central Iran at different extent sizes of variables (0.1, 0.5, 1, 2, and 4 km).
