# Supplementary material for: Combining multiscale niche modeling, landscape connectivity, and gap analysis to prioritize habitats for conservation of striped hyaena (Hyaena hyaena)
Source: PLoS One. 2022 Feb 10;17(2):e0260807. doi: 10.1371/journal.pone.0260807 (PMC8830629; doi:10.1371/journal.pone.0260807)
Supplement: S3 Fig — Binary habitat suitability maps for striped hyaena in central Iran at extent size of 0.1 (a), 0.5 (b), 1 (c), 2 (d) and 4 km (e) using the mean suitability score at occurrence points as threshold. (DOCX) [file pone.0260807.s003.docx]

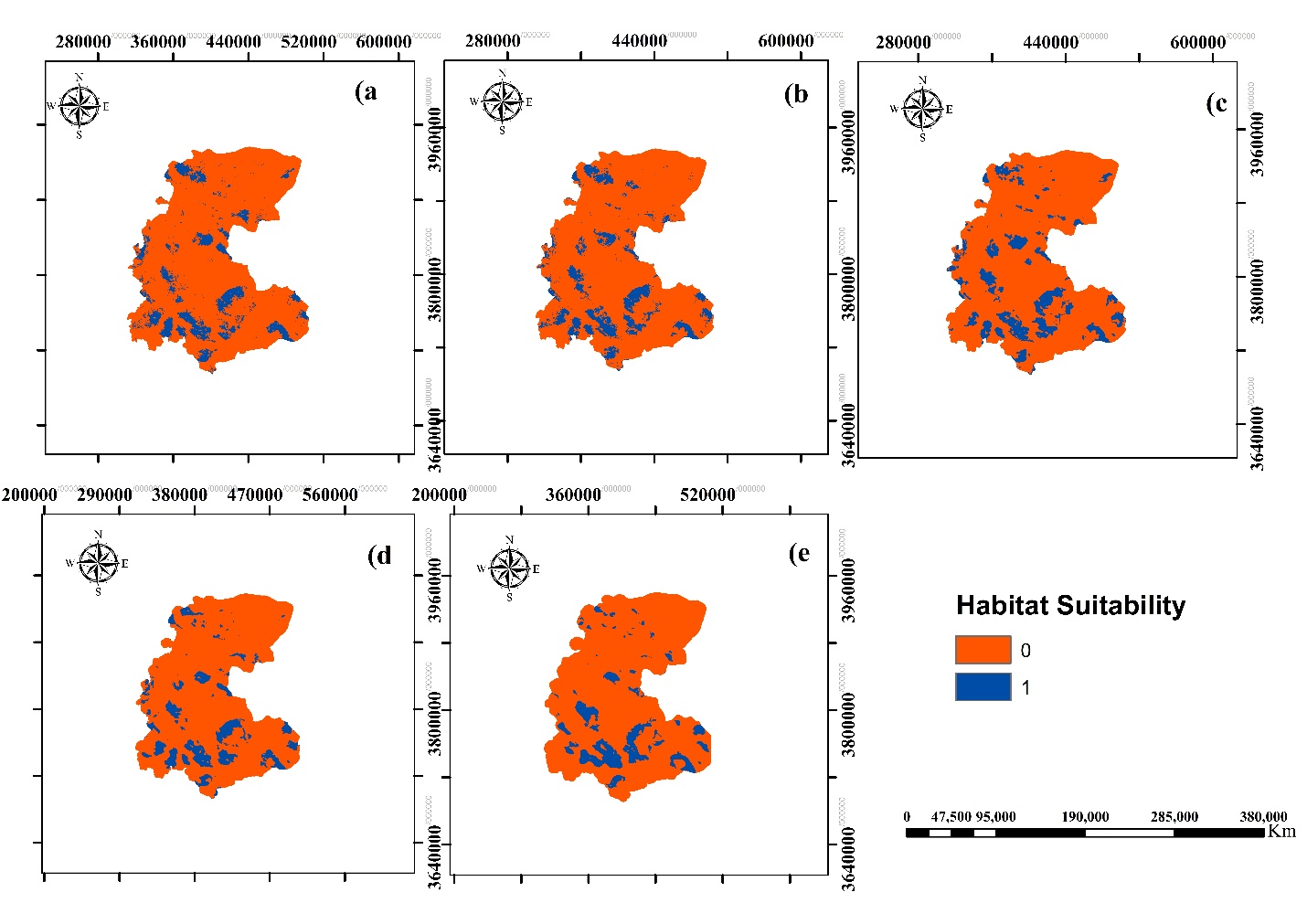


**Fig. S3**. Binary habitat suitability maps for striped hyaena in central Iran at extent size of 0.1 (a), 0.5 (b), 1 (c), 2 (d) and 4 km (e) using the mean suitability score at occurrence points as threshold.
