# Supplementary material for: Combining multiscale niche modeling, landscape connectivity, and gap analysis to prioritize habitats for conservation of striped hyaena (Hyaena hyaena)
Source: PLoS One. 2022 Feb 10;17(2):e0260807. doi: 10.1371/journal.pone.0260807 (PMC8830629; doi:10.1371/journal.pone.0260807)
Supplement: S5 Fig — The colour gradient represents predicted connectivity from weak (blue) to strong (red). (DOCX) [file pone.0260807.s005.docx]

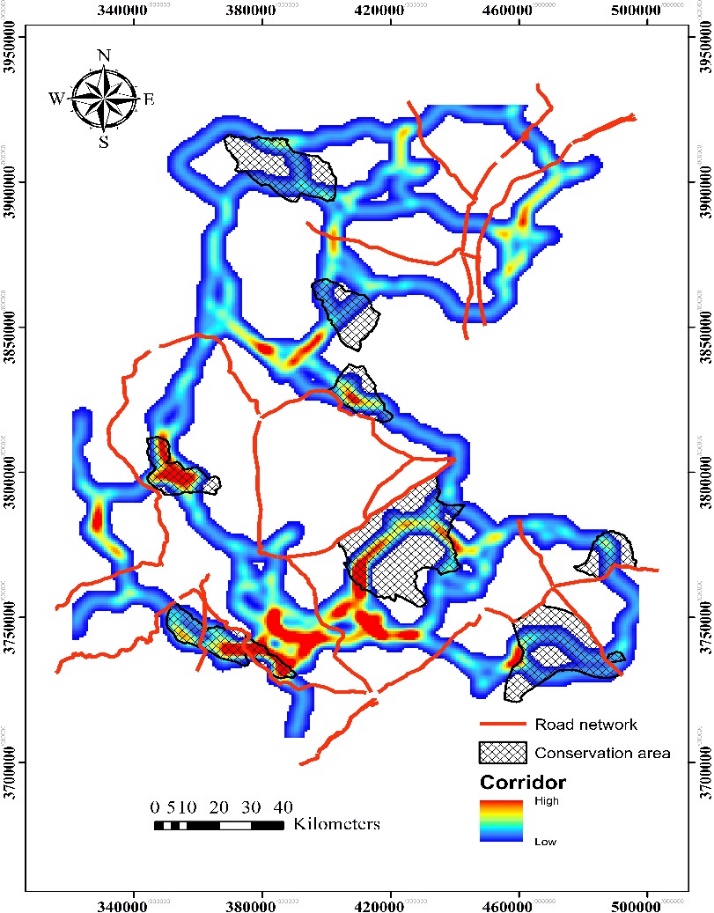


**Fig. S5.** Predicted corridors’ network linking striped hyaena core habitats and conservation areas at variable extent size of 4 km in central Iran. The colour gradient represents predicted connectivity from weak (blue) to strong (red).
