# Supplementary material for: Combining multiscale niche modeling, landscape connectivity, and gap analysis to prioritize habitats for conservation of striped hyaena (Hyaena hyaena)
Source: PLoS One. 2022 Feb 10;17(2):e0260807. doi: 10.1371/journal.pone.0260807 (PMC8830629; doi:10.1371/journal.pone.0260807)
Supplement: S6 Fig — (DOCX) [file pone.0260807.s006.docx]

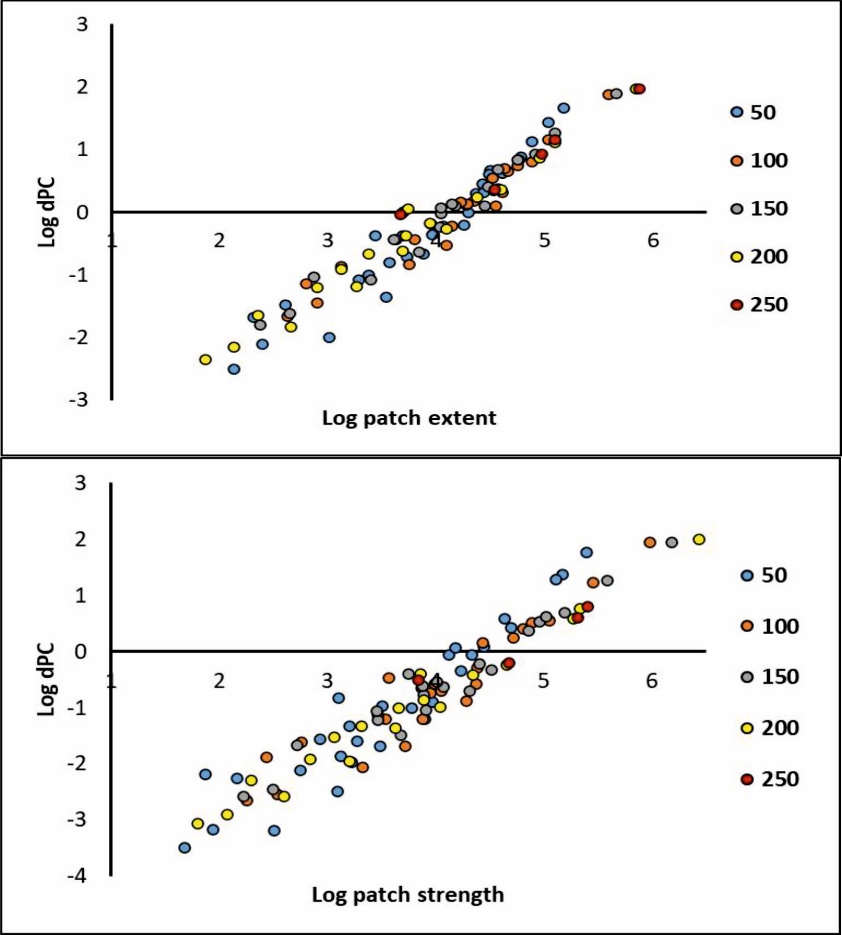


**Fig. S6.** Mean values of delta probability of connectivity (dPC) index calculated for the predicted core habitats of striped hyaena in central Iran based on their extent and strength at five dispersal scenarios.
