# Supplementary material for: Combining multiscale niche modeling, landscape connectivity, and gap analysis to prioritize habitats for conservation of striped hyaena (Hyaena hyaena)
Source: PLoS One. 2022 Feb 10;17(2):e0260807. doi: 10.1371/journal.pone.0260807 (PMC8830629; doi:10.1371/journal.pone.0260807)
Supplement: S1 Table — (DOCX) [file pone.0260807.s007.docx]

**Table S1**: List of the environmental variables used to predict distribution of *H. hyaena* in central Iran.

| **Variable**  **(unit of measurement)** | **Description** | **Source of data** | **Hypothesis** |
| --- | --- | --- | --- |
| Elevation (m) | Digital Elevation Model (DEM) | www.usgs.gov | With increasing elevation, striped hyaena’s habitat suitability decreases. |
| Topographic roughness (m) | The amount of elevation difference between adjacent cells | www.usgs.gov | With increasing topographic roughness, striped hyaena habitat suitability increases to its maximum and then leveled off |
| Density of vegetation types (%) | Density of vegetation types with density ≥ 25% within a moving window of 1 × 1 km | - Iran’s Department of Environment (Personal communication)  - Markazi provincial office of Department of Environment (Personal communication) | Striped hyaena tends to select habitats with intermediate density of vegetation cover |
| NDVI | Normalized Difference Vegetation Index | http://earthexplorer.usgs.gov/ | NDVI increases net primary productivity and therefore increases the probability of occurrence of the species |
| Distance to roads (m) | Euclidean distance to roads | Iranian Forest Ranges and Watershed Management Organization; IFRWO (https://frw.ir/02/Fa/default.aspx) | With increasing road distance, striped hyaena habitat suitability increases due to supplementary sources |
| Distance to human settlements (m) | Euclidean distance to villages | Iranian Forest Ranges and Watershed Management Organization; IFRWO (https://frw.ir/02/Fa/default.aspx) | The presence of human would have negative effects on the probability of occurrence of striped hyaena |
| Distance to dumpsites (m) | Euclidean distance to dumpsites | Markazi provincial office of Department of Environment | Striped hyaenas prefer suitable habitats near dumpsites due to supplementary resources |
| Prey availability | Raster map obtained combining habitat suitability and density maps of the selected prey species | Markazi provincial office of Department of Environment | The presence of prey increases habitats suitability for striped hyaena. |
| Distance to croplands (m) | Euclidean distance to croplands | Markazi provincial office of Department of Environment | Decreasing distance to croplands would decrease striped hyaena habitat suitability. |
| Slope (%) | The rate of change of elevation for each cell | www.usgs.gov | With increasing slop, the probability of suitability will decrease |
