# Supplementary material for: Combining multiscale niche modeling, landscape connectivity, and gap analysis to prioritize habitats for conservation of striped hyaena (Hyaena hyaena)
Source: PLoS One. 2022 Feb 10;17(2):e0260807. doi: 10.1371/journal.pone.0260807 (PMC8830629; doi:10.1371/journal.pone.0260807)
Supplement: S2 Table — (DOCX) [file pone.0260807.s008.docx]

**Table S2**. The area of suitable habitats (km^2^) for striped hyaena covered by the existing conservation areas at five extent sizes of variables in central Iran. PA, protected area; WR, wildlife refuge; NHA, non-hunting area. For the 10-percentile threshold, NHAs provided the highest coverage for the all five extent sizes of variables. However, for the mean threshold, there is a reasonable difference among five extent sizes. In other words, at extent size of 0.1, 0.5, and 4 km NHAs and at extent size of 1 and 2 km PAs had the highest coverage.

| Extent size of variables (km) | | Conservation areas (CAs) | Mean suitability values at the occurrence points | 10^th^ percentile training presence threshold |
| --- | --- | --- | --- | --- |
| 0.1 | PA | | 41194 | 91988 |
|  | WR | | 14108 | 23109 |
|  | NHA | | **51194** | **108622** |
| 0.5 | PA | | 44662 | 96304 |
|  | WR | | 14692 | 96304 |
|  | NHA | | **51981** | **103797** |
| 1 | PA | | **45489** | 93331 |
|  | WR | | 24316 | 24316 |
|  | NHA | | 43285 | **99203** |
| 2 | PA | | **46930** | 97701 |
|  | WR | | 15317 | 23937 |
|  | NHA | | 40639 | **100503** |
| 4 | PA | | 37133 | 96576 |
|  | WR | | 17462 | 25931 |
|  | NHA | | **40342** | **100019** |
