# Supplementary material for: Combining multiscale niche modeling, landscape connectivity, and gap analysis to prioritize habitats for conservation of striped hyaena (Hyaena hyaena)
Source: PLoS One. 2022 Feb 10;17(2):e0260807. doi: 10.1371/journal.pone.0260807 (PMC8830629; doi:10.1371/journal.pone.0260807)
Supplement: S3 Table — (DOCX) [file pone.0260807.s009.docx]

**Table S3**. The most important conservation areas ranked according to the delta probability of connectivity (PC) index and its three fractions (intra, flux, and connector) at the variable extent sizes of 4 km.

| **Rank** | **Conservation category** | **Area name** | **dPC** | **dPC-intra** | **dPC-flux** | **dPC-connector** |
| --- | --- | --- | --- | --- | --- | --- |
| 1 | Non-hunting area | Chal-Khatoon | **52.85** | 7.49 | 31.20 | **14.15** |
| 2 | Wildlife refuge | Rasvand | **44.44** | 10.64 | **33.76** | 0.04 |
| 3 | Protected area | Alvand | **35.09** | 5.89 | 29.17 | 0.02 |
| 4 | Non-hunting area | Palangab | 8.04 | 7.95 | 0.09 | 0 |
| 5 | Wildlife refuge | Jasb | 5.32 | 4.62 | 0.69 | 0 |
| 6 | Non-hunting area | Colahe | 4.76 | 2.83 | 1.89 | 0.03 |
| 7 | Wildlife refuge | Mooteh | 4.22 | 3.43 | 0.78 | 0 |
| 8 | Protected area | Haftad-Gholleh | 4.12 | 3.77 | 0.35 | 0 |
| 9 | Non-hunting area | Bazerjan | 3.48 | 1.64 | 1.82 | 0.02 |
| 10 | Non-hunting area | Kharaghan | 1.82 | 1.76 | 0.05 | 00 |
