# Supplementary material for: Combining multiscale niche modeling, landscape connectivity, and gap analysis to prioritize habitats for conservation of striped hyaena (Hyaena hyaena)
Source: PLoS One. 2022 Feb 10;17(2):e0260807. doi: 10.1371/journal.pone.0260807 (PMC8830629; doi:10.1371/journal.pone.0260807)
Supplement: S4 Table — (DOCX) [file pone.0260807.s010.docx]

**Table 4**. The list of occurrence localities of striped hyaena *(Hyaena hyaena*) used for habitat suitability modeling

| **Presence point No.** | **Longitude** | **Latitude** |
| --- | --- | --- |
| 1 | 353870.2 | 3743309 |
| 2 | 370097.5 | 3740607 |
| 3 | 355608.3 | 3745000 |
| 4 | 384703.3 | 3729666 |
| 5 | 357309.2 | 3742023 |
| 6 | 410709.4 | 3750492 |
| 7 | 403870.5 | 3750939 |
| 8 | 407365.8 | 3755630 |
| 9 | 416325.4 | 3749884 |
| 10 | 464363.1 | 3731480 |
| 11 | 462803.5 | 3741212 |
| 12 | 473884.8 | 3742532 |
| 13 | 389838.3 | 3779854 |
| 14 | 388363.7 | 3777425 |
| 15 | 351178.3 | 3801391 |
| 16 | 358125.4 | 3798487 |
| 17 | 360202.5 | 3798455 |
| 18 | 356138.8 | 3795696 |
| 19 | 330135.3 | 3780962 |
| 20 | 328779.6 | 3782074 |
| 21 | 326282 | 3779829 |
| 22 | 407624.6 | 3830751 |
| 23 | 406051.5 | 3825620 |
| 24 | 407764.8 | 3823279 |
| 25 | 411507.3 | 3824366 |
| 26 | 454920.3 | 3882437 |
| 27 | 452459.5 | 3866065 |
| 28 | 482424.8 | 3905940 |
| 29 | 425173.9 | 3864417 |
| 30 | 420983.3 | 3784229 |
| 31 | 385753 | 3833916 |
| 32 | 402438.9 | 3871916 |
| 33 | 468109.3 | 3776721 |
| 34 | 431879 | 3904861 |
| 35 | 459196.6 | 3926533 |
| 36 | 366116.3 | 3883534 |
| 37 | 384431.8 | 3912321 |
| 38 | 324065.4 | 3818910 |
| 39 | 448065 | 3725383 |
| 40 | 363324.3 | 3863872 |
| 41 | 487294.9 | 3774358 |
| 42 | 322050.2 | 3745894 |
| 43 | 353991.3 | 3815761 |
| 44 | 446445.5 | 3772939 |
| 45 | 390154.5 | 3708914 |
| 46 | 397969.3 | 3846477 |
| 47 | 382852.9 | 3751795 |
| 48 | 393419.4 | 3734112 |
| 49 | 372475.8 | 3753985 |
| 50 | 420819.6 | 3741763 |
| 51 | 446564.1 | 3759568 |
| 52 | 360443.2 | 3784775 |
| 53 | 367727.5 | 3734870 |
| 54 | 469787.6 | 3881476 |
