# Supplementary material for: Combining multiscale niche modeling, landscape connectivity, and gap analysis to prioritize habitats for conservation of striped hyaena (Hyaena hyaena)
Source: PLoS One. 2022 Feb 10;17(2):e0260807. doi: 10.1371/journal.pone.0260807 (PMC8830629; doi:10.1371/journal.pone.0260807)
Supplement: S1 Text — (DOCX) [file pone.0260807.s011.docx]

**Text S1**. Predicting the distribution of the prey species

We modeled distribution of the prey species using the maximum entropy algorithm (MaxEnt). We first obtained 139, 40 and 174 occurrence points for wild goat (*Capra aegagrus*), goitered gazelle (*Gazella subguturosa*), and mouflon (*Ovis gmelini*), respectively collected from data between 2015 to 2018 from Markazi Department of Environment and also the data used by Karami et al, (2020). These localities were obtained from a variety of sources including opportunistic direct observation of denning sites, scat identification, and direct sightings.

To avoid using spatially biased occurrence data, each group of the occurrence points was tested for spatial autocorrelation using Moran’s I test. For the three species we selected nine environmental and anthropogenic variables to build the distribution model, including slope, elevation, topographic roughness, aspect, distance to roads, distance to human settlements, distance to croplands, density of vegetation types and NDVI. To test for multicollinearity among these variables, we calculated Pearson’s correlation coefficient, and found a high correlation between two variables of slope and topographic roughness (correlation more than 0.8). To select between these two variables for each prey species, we ran MaxEnt using all variables, including both slope and topographic roughness. Then, based on the results of jackknife test of variable importance, we selected the topographic variable with higher contribution to the model (topographic roughness for goitered gazelle and wild goat, and slope for mouflon). For each prey species, the selected set of variables was then used to build the final distribution model. The MaxEnt models were run with ten replicates, 10,000 random background points and 500 iterations. We used the 75% of occurrence points as training data to calibrate the models, and the remaining 25% as test data to evaluate models’ predictions.
