# Supplementary material for: Combining multiscale niche modeling, landscape connectivity, and gap analysis to prioritize habitats for conservation of striped hyaena (Hyaena hyaena)
Source: PLoS One. 2022 Feb 10;17(2):e0260807. doi: 10.1371/journal.pone.0260807 (PMC8830629; doi:10.1371/journal.pone.0260807)
Supplement: S2 Text — (DOCX) [file pone.0260807.s012.docx]

**Text S2**. The impact of distance to roads was found to be positive on habitat suitability for striped hyaena in central Iran up to a certain distance of nearly 1 km; however, with increasing distance above that, the probability of occurrence decreased (see response curves in Fig. S2). Elevation was the second top variable in predictions made at four spatial scales except for the extent size of 0.1 km where it was replaced by the distance to croplands. The response curve of this variable indicated a negative relationship meaning that areas far away from croplands were identified to have higher suitability for the species. Distance to dumpsites also gained high contribution at three extent sizes of 0.1, 2 and 4 km with a positive relationship up to a certain distance of 2 km and negative afterwards (see response curves). Among the environmental variables, density of vegetation types and NDVI had the least impotence in predicting striped hyaena distribution across the study area. However, according to the associated response curve, the striped hyaena was predicted to respond positively to increasing density of vegetation types up to a certain value after which the species was less likely to occur across more dense habitats.
