# Supplementary material for: Combining multiscale niche modeling, landscape connectivity, and gap analysis to prioritize habitats for conservation of striped hyaena (Hyaena hyaena)
Source: PLoS One. 2022 Feb 10;17(2):e0260807. doi: 10.1371/journal.pone.0260807 (PMC8830629; doi:10.1371/journal.pone.0260807)
Supplement: S3 Text — (DOCX) [file pone.0260807.s013.docx]

**Text S3**. At dispersal ability of 100 km there were five large habitat patches which were located in the south, west, and eastern parts of the landscape in central Iran (patches 21, 16, 22, 11 and 24). The number of recognized patches for dispersal ability of 150 km was five large patches (22, 15, 21, 17 and 11). There was only one large patch at dispersal ability of 200 and 250 km which were located in south parts of the landscape (200 km (patch 18) and 250 km (patch 5). These recognized large core habitats covered by three conservation areas (Haftad-Gholleh, Alvand, and Rasvand).
